# Supplementary material for: Hyperbaric oxygen therapy improves clinical symptoms and functional capacity and modulates thalamic connectivity in ME/CFS: a prospective cohort study
Source: J Transl Med. 2026 Jun 5;24:744. doi: 10.1186/s12967-026-08324-6 (PMC13244963; doi:10.1186/s12967-026-08324-6)
Supplement: Supplementary file 1 — Supplementary material 1 [file 12967_2026_8324_MOESM1_ESM.docx]

**Supplementary Material S1**


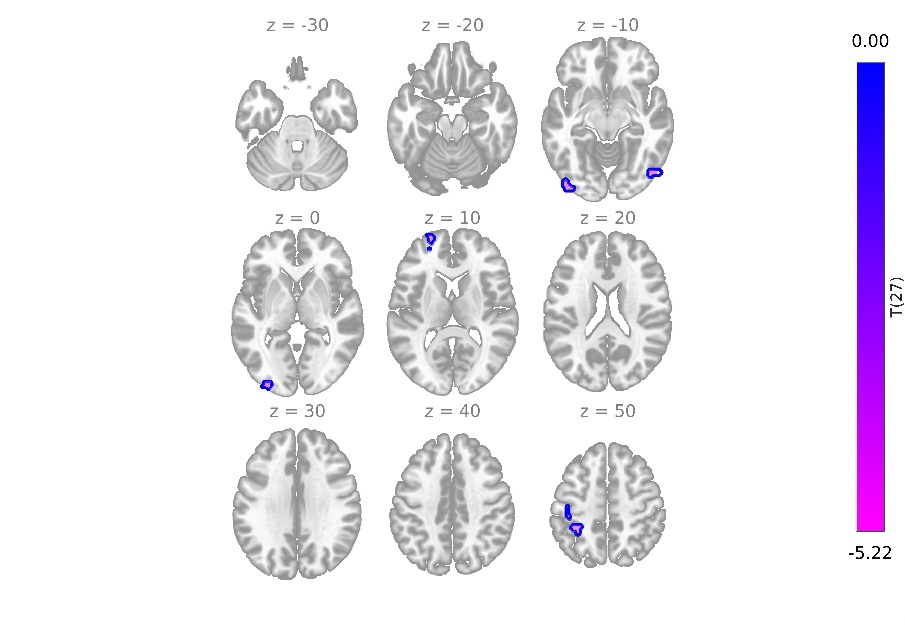


**Figure S1 (Sensitivity analysis):** Thalamic functional connectivity in ME/CFS patients pre-vs-post-treatment (Threshold: voxel-wise p < 0.001; cluster-level p < 0.05, FDR-corrected).

Patients longitudinal (pre-vs-post-treatment), seed: left thalamic sensorimotor cluster. Post-treatment reductions in thalamic connectivity were observed with left sensorimotor regions (precentral and postcentral gyri), and bilateral visuo-occipital regions (lateral occipital cortex, occipital pole).
